# miRNA in cardiac development and regeneration

**DOI:** 10.1186/s13619-021-00077-5

**Published:** 2021-06-01

**Authors:** Zhaohui Ouyang, Ke Wei

**Affiliations:** Institute for Regenerative Medicine, Shanghai East Hospital, Shanghai Institute of Stem Cell Research and Clinical Translation, Shanghai Key Laboratory of Signaling and Disease Research, Frontier Science Center for Stem Cell Research, School of Life Sciences and Technology, Tongji University, Shanghai, 200092 P.R. China

**Keywords:** microRNA, heart, cardiomyocyte, development, proliferation, regeneration

## Abstract

Ischemic heart disease is one of the main causes of morbidity and mortality in the world. In adult mammalian hearts, most cardiomyocytes are terminally differentiated and have extremely limited capacity of proliferation, making it impossible to regenerate the heart after injuries such as myocardial infarction. MicroRNAs (miRNAs), a class of non-coding single-stranded RNA, which are involved in mRNA silencing and the regulation of post-transcriptional gene expression, have been shown to play a crucial role in cardiac development and cardiomyocyte proliferation. Muscle specific miRNAs such as miR-1 are key regulators of cardiomyocyte maturation and growth, while miR-199-3p and other miRNAs display potent activity to induce proliferation of cardiomyocytes. Given their small size and relative pleiotropic effects, miRNAs have gained significant attraction as promising therapeutic targets or tools in cardiac regeneration. Increasing number of studies demonstrated that overexpression or inhibition of specific miRNAs could induce cardiomyocyte proliferation and cardiac regeneration. Some common targets of pro-proliferation miRNAs, such as the Hippo-Yap signaling pathway, were identified in multiple species, highlighting the power of miRNAs as probes to dissect core regulators of biological processes. A number of miRNAs have been shown to improve heart function after myocardial infarction in mice, and one trial in swine also demonstrated promising outcomes. However, technical difficulties, especially in delivery methods, and adverse effects, such as uncontrolled proliferation, remain. In this review, we summarize the recent progress in miRNA research in cardiac development and regeneration, examine the mechanisms of miRNA regulating cardiomyocyte proliferation, and discuss its potential as a new strategy for cardiac regeneration therapy.

## Background

As one of the most important organs, the heart is the engine of the blood flow in our body, pushes blood through every organ and tissue to supply oxygen and various nutrients. Ischemic heart disease, especially myocardial infarction (MI), is one of the leading causes of death in both developing and developed countries (Collaborators 2018), and generates enormous personal, social and economic burden worldwide (Roth et al. 2017). Massive cardiomyocyte death after MI often leads to irreversible deterioration of cardiac physiology and eventually heart failure and death. The underlying reason is that the adult mammalian hearts have extremely limited regenerative capacity (Eschenhagen et al. 2017), making most patients with myocardial infarction deteriorate toward heart failure and no cure is available except heart transplantation. Over a century, our understanding had been that cardiomyocytes in the adult mammalian heart were terminally differentiated and did not have any regenerative capacity (Porrello and Olson 2014). However, recent advances in basic research, especially in the 21st century, challenged this dogma. First, adult zebrafish heart was found to be able to fully regeneration after injury via proliferation of existing cardiomyocytes (Poss et al. 2002), and other lower vertebrates, like the newts (Witman et al. 2011), showed similar capacity to regenerate heart after injury. In addition, using radioactive labeling enabled by atmosphere nuclear test, cardiomyocytes renewal in human was calculated to be around 1% per year at age 20, and gradually declined to 0.3% per year at age 75 (Bergmann et al. 2009). Even more encouragingly, neonatal mice were found to retain a robust capacity for cardiac regeneration until postnatal day 7 (Porrello et al. 2011b), and such activity is conserved in other mammals such as swine (Ye et al. 2018). These new findings indicated that stimulating cardiac regeneration might be a rational strategy for treating patients with ischemic heart diseases.

Several promising strategies have been proposed and tested for heart regeneration. The decades-long endeavor in searching for endogenous cardiac stem cells with myogenic capacity was plagued with research misconduct (Kaiser 2018), and is coming to an end with no evidence of non-myocyte to myocyte transition observed in adult mouse heart (Li et al. 2018b). Injecting cardiomyocytes derived from embryonic stem cells (ESCs) or inducible pluripotent stem cells (iPSCs) to replace the lost cardiomyocytes has the advantage of unlimited supply of cardiomyocytes, while facing the challenge of the unwanted side effect of causing arrythmia (Chong et al. 2014; Shiba et al. 2016). Reprogramming the non-cardiomyocytes to the cardiomyocytes avoids using exogenous cells (Qian and Srivastava 2013), but concerns over the safety and efficacy of the reprogramming agents remain (Sadek and Olson 2020). As proliferation of existing cardiomyocytes was found to be the underlying mechanism of the regeneration of zebrafish (Poss et al. 2002) and neonatal mouse heart (Porrello et al. 2011b), promoting cardiomyocytes to re-enter the cell cycle and proliferate may be the ultimate path to activate the endogenous regenerative capacity. However, it requires comprehensive understanding of the fundamental mechanism of cardiomyocytes proliferation and regeneration (Sadek and Olson 2020), which may offer novel insights for designing effective and safe therapies to treat ischemic heart disease.

A large body of research has shed light on the molecular mechanisms regulating cardiomyocyte proliferation. For example, core cell cycle proteins such as Cyclin D2 (Pasumarthi et al. 2005), as well as cyclin-dependent kinase 1 (CDK1), CDK4, cyclin B1, and cyclin D1 (Rojas et al. 2008; Mohamed et al. 2018), and nuclear proteins regulating the cell cycle, such as Gata4 (Rojas et al. 2008), Meis1 (Mahmoud et al. 2013), and Hand2 (Schindler et al. 2014) have been shown to directly regulate cardiomyocyte proliferation. Signaling pathways such as Notch pathway (Campa et al. 2008), ErbB2/4 pathway (Bersell et al. 2009; Gemberling et al. 2015) and Hippo pathway (Heallen et al. 2011; Xin et al. 2013; Heallen et al. 2013), as well as p38 kinase pathway (Engel et al. 2005), have also been implicated in regulating cardiomyocyte proliferation. Exogenous stimulations such as hypoxia (Kimura et al. 2015), and extracellular ligands such as Fgf1 (Engel et al. 2005), Notch1 (Campa et al. 2008), Nrg1 (Bersell et al. 2009), Fstl1 (Wei et al. 2015), and Agrin (Bassat et al. 2017) were shown to promote cardiomyocyte proliferation, while thyroid hormone (Hirose et al. 2019) were found to negatively regulate this process.

Recently, growing amount of evidence has demonstrated that microRNAs (miRNAs), small non-coding RNA which regulate post-translational expression of their target genes, play key roles in cardiac development, cardiomyocyte proliferation and heart regeneration (Braga et al. 2020). Unlike many of the aforementioned factors promoting cardiomyocyte proliferation, which are difficult to target or delivery, or having severe side effects, miRNAs are small (~22 nucleotides), enabling relatively easy delivery, and target multiple genes to exert pleiotropic functions, which may maximize their beneficial effects of the treatment. Therefore, miRNA has emerged as a promising tool for therapies stimulating cardiac regeneration. In this review, we discuss the functions of miRNA in the heart, especially during development and regeneration, and explore the possibility of using miRNA as new strategies for cardiac regeneration therapy.

## Main Text

### Biogenesis and Function of microRNA

MicroRNA (miRNA) are a class of non-coding single-stranded RNA (containing about 22 nucleotides) encoded by endogenous genes, which are involved in RNA silencing and the regulation of post-transcriptional gene expression in animals, plants, and even some prokaryotes (Bartel 2004; Ambros 2004). miRNAs inhibit the expression of targeted genes by binding with the targeted mRNA, mostly in the 3’UTR. Each miRNA may have multiple target genes and the same gene may also be regulated by several miRNAs, forming a complex regulatory network. Since the first miRNA (*lin-4*) was discovered in the *Caenorhabditis elegans* in the early 1990s (Lee et al. 1993), thousands of miRNAs have been identified in almost all species during the past 30 years, including about 2300 mature miRNAs discovered in humans (Alles et al. 2019). More importantly, some microRNAs, such as *let-7*, have been detected in a variety of animals, including some invertebrates, suggesting that miRNA is evolutionarily conserved and has a general biological role (Pasquinelli et al. 2000).

The basic process for the formation of mature miRNAs in animals is highly conserved: 1) Primary miRNA transcription product (abbreviated as pri-miRNA) is transcribed by RNA polymerase II (Lee et al. 2004). The length of pri-miRNA ranges from 300 to 10 kbp, with an imperfectly matched stem-ring hairpin structure in the middle (Cai et al. 2004). 2) pri-miRNA hairpin is cut by Drosha endonuclease and generates pre-miRNA (a ~60 nt stem-loop) (Ha and Kim 2014; Nguyen et al. 2015). Drosha is a class 2 RNase III enzyme, which cut the 3'-end and 5'-end of the pri-miRNA hairpin with a 2-bp offset and generate a pre-miRNA, which retains the stem-loop (Lee et al. 2003). 3) The pre-miRNA is then transported out of the nucleus and into the cytosol via Exportin 5/RanGTP (Lund et al. 2004). The pre-miRNA is further processed by Dicer, an endonuclease which cuts off the loop region of the pre-miRNA, and liberates the stem, a miRNA duplex (named miRNA-miRNA*), containing two largely complimentary miRNAs. The miRNA duplex is then loaded into an Argonaute protein (Ago) to form pre-RNA-induced silencing complex (pre-RISC) (Iwasaki et al. 2010). At this stage, one strand will be degraded and the other strand becomes the mature single-stranded miRNA (~22nt) (Hutvágner et al. 2001), and the mature miRNA and Ago constitute the RISC complex to degrade target mRNAs (Kawamata and Tomari 2010). Targets were determined by complementation of the seed region as few as 6–8 nucleotides at the 5' end of the miRNA to the sequence of the mRNA, especially in its 3’-UTR. Thus, one gene can be targeted by multiple miRNAs, and one miRNA can targets multiple genes (Bartel 2018). After binding with their targets, microRNAs can downregulate gene expression by either of the two posttranscriptional silencing mechanisms (Bartel 2004). The RISC on the target mRNA either degrades the targeted mRNA, or represses translation without mRNA degradation (Bartel 2004). Numerous studies have demonstrated that miRNAs play important roles in many biological processes, including embryonic development (Ambros 2011), haematopoiesis (Wang et al. 2014), apoptosis (Thompson and Cohen 2006), proliferation (Thompson and Cohen 2006) and so on. Without exception, miRNAs have been found to modulate cardiac development and regeneration, and have been considered as a tool to treat cardiac diseases (Braga et al. 2020).

### miRNA and heart development

The overall importance and requirement of the miRNA system in the heart were demonstrated as Dicer, the endonuclease processing the maturation of miRNAs, was found to be critical for cardiac development and growth (Zhao et al. 2007; Chen et al. 2008; da Costa Martins et al. 2008; Saxena and Tabin 2010). Global *Dicer* knockout displayed early embryonic lethality before cardiogenesis (Bernstein et al. 2003). To examine the function of *Dicer* in cardiac development, Zhao et al. deleted *Dicer* specifically during early cardiogenesis using a cardiac specific *Nkx2.5-Cre* (Zhao et al. 2007). The resulting loss-of-function of *Dicer* in embryonic heart caused lethality at embryonic day 12.5 (E12.5) due to cardiac failure (Zhao et al. 2007), revealing the requirement of the miRNA system for proper development of the heart. Using a different *Nkx2.5-Cre* allele, Saxena and Tabin observed ventricular septal defect after deleting *Dicer* in the developing murine heart (Saxena and Tabin 2010), with mutant embryos living up to E13.75 (Saxena and Tabin 2010), slightly longer than the previous report (Zhao et al. 2007). To assess the role of miRNAs in late development and postnatal heart, Chen et al. used a relatively late cardiac specific Cre line, *MHC*^*Cre/+*^, to delete *Dicer*, and found that *Dicer* mutant mice suffered from severe dilated cardiomyopathy and heart failure, and died shortly after birth (Chen et al. 2008). Similarly, induced deletion of *Dicer* in postnatal cardiomyocytes usin*g αMHC-MerCreMer in 3*-week-old mice resulted in mild ventricular remodeling, and the juvenile mutant mice died within one week after cardiac-restricted *Dicer* deletion (da Costa Martins et al. 2008). Overall, in all stages of cardiac development and growth, loss-of-function of *Dicer* consistently led to cardiac dysfunction and death, emphasizing the crucial requirement of a functional miRNA system in the heart.

Specific miRNAs are evolutionally conserved and enriched in particular organs, and these organ- or tissue-specific expression of miRNAs participate in tissue specification, cell lineage decisions, as well as tissue homeostasis (Lagos-Quintana et al. 2002) (Sempere et al. 2004). Identification of miRNAs specifically expressed in the heart, was the first step in revealing heart specific functions of these miRNAs. miR-1 was initially identified as the most abundant miRNA in the rodent heart (Lagos-Quintana et al. 2002), accounting for an astonishing amount (45%) of all miRNAs in the heart (Lagos-Quintana et al. 2002). In 2004, a broader and deeper screen detected 30 organ-specific and organ-enriched miRNAs by analyzing the expression of 119 miRNAs in adult organs from mouse and human, and discovered six miRNAs specifically expressed or enriched in skeletal muscle and heart: miR-1b, -1d, -133 and -206, miR-143 and -208 (Sempere et al. 2004), suggesting that they might carry muscle or cardiac specific functions. The majority of these miRNAs and a few other miRNAs are later confirmed as enriched in developing heart (miR-1 (Zhao et al. 2005), miR-133 (Liu et al. 2007), and miR-208b (Callis et al. 2009)), and postnatal heart (miR-208a (Callis et al. 2009) and miR-499 (van Rooij et al. 2009)). These muscle specific miRNAs were termed MyomiRs (McCarthy 2008), which play significant role in cardiac and muscle development .

The muscle specific miR-1 and miR-133 are transcribed separately in invertebrates, whereas they are found to be genetically connected in vertebrates (Chen et al. 2006). In mouse, two miR-1s (miR-1-1 and miR-1-2, with identical sequences) and their related miR-133s (miR-133a-2 and miR-133a-1, respectively) are clustered together on mouse chromosome 2 and chromosome 18, with each pair transcribed as a single bicistronic transcript, and generated two independent miRNAs (Zhao et al. 2005; Chen et al. 2006). While in human, miR-133a-1, miR-133a-2 and miR-133b are 3 known miR-133 genes in the genome, which are clustered with miR-1-2, miR-1-1 and miR-206 on chromosomes 18, 20 and 6, respectively (Ivey et al. 2008). The expression of miR-1 and miR-133 in the embryonic heart is regulated by the transcription factors SRF, MEF2 and myogenic regulatory factor (MRF), MyoD (Zhao et al. 2005; Ivey et al. 2008).

miR-1, the first miRNA reported to be enriched in heart (Lagos-Quintana et al. 2002), was found to play a pivotal role in development of cardiac muscle, as its overexpression in the heart led to decreased cardiomyocyte proliferation and thin myocardium, as well as deceased expression of important transcription factors like *Hand2* (Zhao et al. 2005). As miR-1-1 and miR-1-2 share the same sequence, their individual functions can only be dissected through loss-of-function experiments. Zhao et al. first deleted *miR-1-2* in mice, and found cardiac morphogenetic defects, including ventricular septal defects, increased cardiomyocyte proliferation, as well as *Hand2* overexpression, in *miR-1-2* mutants, suggesting that miRNA-1-2 participates in regulating cardiac morphogenesis, cardiac electrophysiology, and cardiac cell cycle (Zhao et al. 2007). Heidersbach and colleagues deleted *miR-1-1* and *miR-1-2* separately, and generated compound *miR-1* knockout mice, which exhibit embryonic lethality due to sarcomere disruption (Heidersbach et al. 2013), suggesting miR-1 may regulate sarcomere formation in the mammalian heart. miRNAs are small and often reside within introns of genes, which makes it difficult to keep the host and/or surrounding genes intact when knocking out miRNA (Wei et al. 2014). *miR-1-2* happens to locate within *Mib1* gene, and the two aforementioned *miR-1-2* knockout approaches left selection cassette in the targeted allele (Zhao et al. 2007; Heidersbach et al. 2013), making the interpretation of the phenotypes complicated. Zhao’s group further created a miR-1 double knockout (miR-1 dKO) mice with minimal remnant of exogenous component (Wei et al. 2014), which showed dilated cardiomyopathy and lethality before P17, with altered proliferation, glycolysis, glycogenesis, and sarcomeric structures (Wei et al. 2014), while lacking more severe phenotypes of ventricular septal defects in previous miR-1 knockout mice (Zhao et al. 2007; Heidersbach et al. 2013). The combination of these results suggest miR-1 is critical for cardiac development and cardiomyocyte maturation, and suggested that approach with precision is required for genetic manipulation of endogenous miRNAs.

As the muscle-specific miRNA linked to miR-1, miR-133 is also involved in the regulation of cardiac development. Both miR-133a-1 and miR-133a-2 knockout mice are normal (Liu et al. 2008), whereas miR-133a double-knockout embryonic heart showed increase cardiomyocyte proliferation as well as apoptosis, disrupted sarcomere structure and dysregulated expression of smooth muscle genes (Liu et al. 2008), with nearly half of the double-mutant mice died due to ventricular septal defects before weaning (Liu et al. 2008). As miR-1 and miR-133 are clustered together, it is interesting to see if the two bicistronic clusters (miR-1/miR-133a-2 and miR-1-2/miR-133a-1) are functionally district or redundant. The mice lacking either miR-1-1/133a-2 or miR-1-2/133a-1 cluster can survive without major cardiac developmental defects, suggesting the functions between the two clusters are largely redundant (Wystub et al. 2013). However, loss of both miR-1/133 clusters leads to early embryonic lethality before E11.5 due to aberrant heart development (Wystub et al. 2013), more severe than miR-1s double knockout (Wei et al. 2014) and miR-133s double knockout (Liu et al. 2008). Mechanistic study suggested that their cooperative suppression of *Myocardin* gene expression underlies their function of promoting maturation of cardiomyocytes (Wystub et al. 2013). Overall, these findings reveal critical roles for miR-1 and miR-133 in orchestrating cardiac development, while miR-1 and miR-133a regulate these biological processed in a functionally redundant manner.

As producing functional cardiomyocytes from ES/iPSCs gains attention as therapeutic options to treat heart disease, the role of MyomiRs in cardiomyocyte differentiation from pluripotent stem cells has also been investigated. miR-1 and miR-133 were found to be enriched in early precardiac mesoderm during differentiation of mouse and human embryonic stem cells (Ivey et al. 2008), and both of them can promote mesoderm differentiation and suppress endoderm differentiation (Ivey et al. 2008). Remarkably, the role of miR-1 and miR-133 in specification of mesodermal cell is opposite: miR-1 promotes the cardiac progenitor cells to exit from the cell cycle and differentiates into cardiac muscle, while miR-133 inhibits the differentiation of ES cells toward a cardiac fate (Ivey et al. 2008). Notch ligand Delta-like 1 (Dll-1) was found to be an effective target of miR-1 (Ivey et al. 2008), which is consistent with the *in vivo* study in *Drosophila*, in which Kwon et al. demonstrated that *Drosophila miR-1* (*dmiR-1*) is involved in cardiac differentiation by targeting the Notch ligand *Delta* (Kwon et al. 2005). Glass and Singla transplanted the embryonic stem cells transfected with miR-1 into the infarcted mice heart, and found that miR-1 may drive cardiac differentiation, inhibit apoptosis and improve heart function via regulating the PTEN/Akt pathway (Glass and Singla 2011). miR-1 was also found to promote cardiomyocyte commitment in human ES cell derived-multipotent cardiovascular progenitors by suppressing both WNT and FGF signaling pathways (Lu et al. 2013).

Besides miR-1 and miR-133, miR-499 also plays important role in cardiac differentiation in human ES cells. miRNA profiling of human embryonic stem cells and human embryonic stem cells derived cardiomyocytes (hESC-CMs) have showed that the cardiac-specific miR-499 is highly expressed in hESC-CMs, and its overexpression enhanced cardiac gene expression (Wilson et al. 2010). miR-499 was also found to be a crucial regulator of ventricular specification by enhancing the expression of β-MHC and exerting multiple effects on electrophysiology in hESC-CMs (Fu et al. 2011).

In addition to their role in cardiac development and cardiomyocyte differentiation, miRNAs also play important and complex role in postnatal and adult heart. The importance of Dicer and the overall miRNA system in the adult mouse heart were shown by its mutant causing severe hypertrophy and dilated cardiomyopathy (da Costa Martins et al. 2008). And one great example of specific miRNAs important for postnatal heart is the family of three miRNAs, miRNA-208a, miRNA-208b and miRNA-499, locating in introns of *Myh6*, *Myh7* and *Myh7b* genes, respectively (Chistiakov et al. 2016). In mammals, both α-myosin heavy chain (α-MHC, the faster contracting isoform, encoded by *Myh6* gene) and β-myosin heavy chain (β-MHC, the slower contracting isoform, encoded by *Myh7* gene) are main functional proteins carrying out cardiomyocyte contraction (Lompré et al. 1984), and their expression are under tight control during development and aging, while heart diseases are often accompanied by dysregulation of these two genes (Gupta 2007). And this family of miRNAs located within the *Myh6* and *Myh7* genes, were found to regulate the expression of their host genes and are important for cardiac growth and stress response (van Rooij et al. 2007; van Rooij et al. 2009). miR-208a, located within *Myh6* gene, was found to be necessary for stress-dependent *Myh7* expression and cardiac growth (van Rooij et al. 2007). Mechanistically, it negatively regulates thyroid hormone receptor (TR) coregulator TR-associated protein 1 (THRAP1), and thus controls the thyroid hormone responsiveness and pathological expression of *Myh7* (van Rooij et al. 2007). miR-208a is required for up-regulation of *Myh7* and repression of fast muscle genes (van Rooij et al. 2009), while miR-208b and miR-499 redundantly play a dominant role in response to stress and hypothyroidism (van Rooij et al. 2009). There are numbers of miRNAs that have been shown to be critical to maintain cardiac homeostasis in adults, and their dysregulation is associated with cardiac diseases especially hypertrophy (Barwari et al. 2016). Details of the function of these miRNAs in adult heart can be found in other great reviews (Barwari et al. 2016; Poller et al. 2018), as they are beyond the scope of this review, in which we focus on cardiac development and regeneration.

Other than miRNAs specific or enriched in the heart, some miRNAs, which have fundamental cellular functions and are expressed more broadly, also regulate cardiogenesis. The best example is the miR-17-92 cluster, originally discovery in cancer for its oncogenic function (He et al. 2005). Global knockout of the miR-17-92 cluster caused neonatal lethality due to circulatory failure with lung hypoplasia and cardiac septal defect (Ventura et al. 2008). Isl1 and Tbx1, transcription factors important for cardiac development, were identified as direct targets of miR-17-92 (Wang et al. 2010). Cardiac specific knockout of the cluster led to proliferation defects in cardiomyocyte, suggesting miR-17-92 is necessary for proper cardiomyocyte proliferation during development (Chen et al. 2013).

Additional evidence of miRNA playing roles in cardiac development came from zebrafish, which has transparent embryos easy for observation and a range of tools available for genetic manipulation. Null mutant of *dicer* in zebrafish showed developmental arrest (Wienholds et al. 2003), and overall morphogenesis defects, including cardiac chamber specification defects, without affect early cardiomyocyte differentiation (Giraldez et al. 2005). The global embryonic defect, yet less severe than the early lethal phenotype of *Dicer* null mutant mouse embryos (Bernstein et al. 2003) indicates the functions of Dicer and overall microRNA system are conserved in vertebrates with limited degree of divergency. Interestingly, miR-1 and miR-133 in zebrafish were found to regulate development of all striated muscle (Mishima et al. 2009), by targeting genes encoding actin-related and actin-binding proteins, thus facilitating sarcomeric organization (Mishima et al. 2009). The robust development of zebrafish which does not depend on proper cardiac function may explain the difference between the obvious cardiac phenotypes of mouse mutant of miR-1 (Wei et al. 2014) and miR-133 (Wystub et al. 2013), and the pan-striated muscle defects in zebrafish (Mishima et al. 2009). There are also miRNAs, not been studied or showed no effects in cardiac development in mouse, that were found to regulate zebrafish cardiac development. For example, disruption of miR-138 by morpholino (Nasevicius and Ekker 2000) led to cardiogenic defects with altered cardiomyocyte morphology and cardiac function, together with disrupted retinoic acid signaling (Morton et al. 2008). miR-218, which is located in introns of *slit2/3* genes in zebrafish, was found to be strongly expressed in developing heart (Fish et al. 2011). And its knockdown led to defects in cardiac progenitors’ migration and heart tube formation (Fish et al. 2011; Chiavacci et al. 2012). The Slit/Robo pathway regulating cell movement is shown to be regulated by miR-218, thus controlling cardiac cell migration (Fish et al. 2011). Without knockout models in mouse, the role of miR-138 and miR-218 in mammalian heart development is currently unknown. However, the negative role of miR-218 in cardiomyocyte differentiation from mouse ESCs (Xu et al. 2019) may provide hints that miR-218 might also play a role in mammalian cardiac development. miR-143, known for its role in mammalian smooth muscle cells differentiation (Cordes et al. 2009) and phenotypic switch in response to injury (Vacante et al. 2019), was found to be essential for zebrafish cardiac chamber morphogenesis (Deacon et al. 2010), as its inhibition by morpholino led to cardiac malformation and defects in growth and elongation of developing cardiomyocytes (Deacon et al. 2010). Adducin3, which is a cytoskeleton component important for cell-cell contact, was shown to be directly targeted by miR-143 (Deacon et al. 2010). With its crucial role in mammalian smooth muscles and no obvious cardiac phenotype in its knockout mouse (Xin et al. 2009), the discrepancy in zebrafish and mouse models highlights the possible divergent expression and function of miRNAs in different species, and calls for caution in interpreting results of miRNA manipulation in different animal models.

**Fig. 1:**
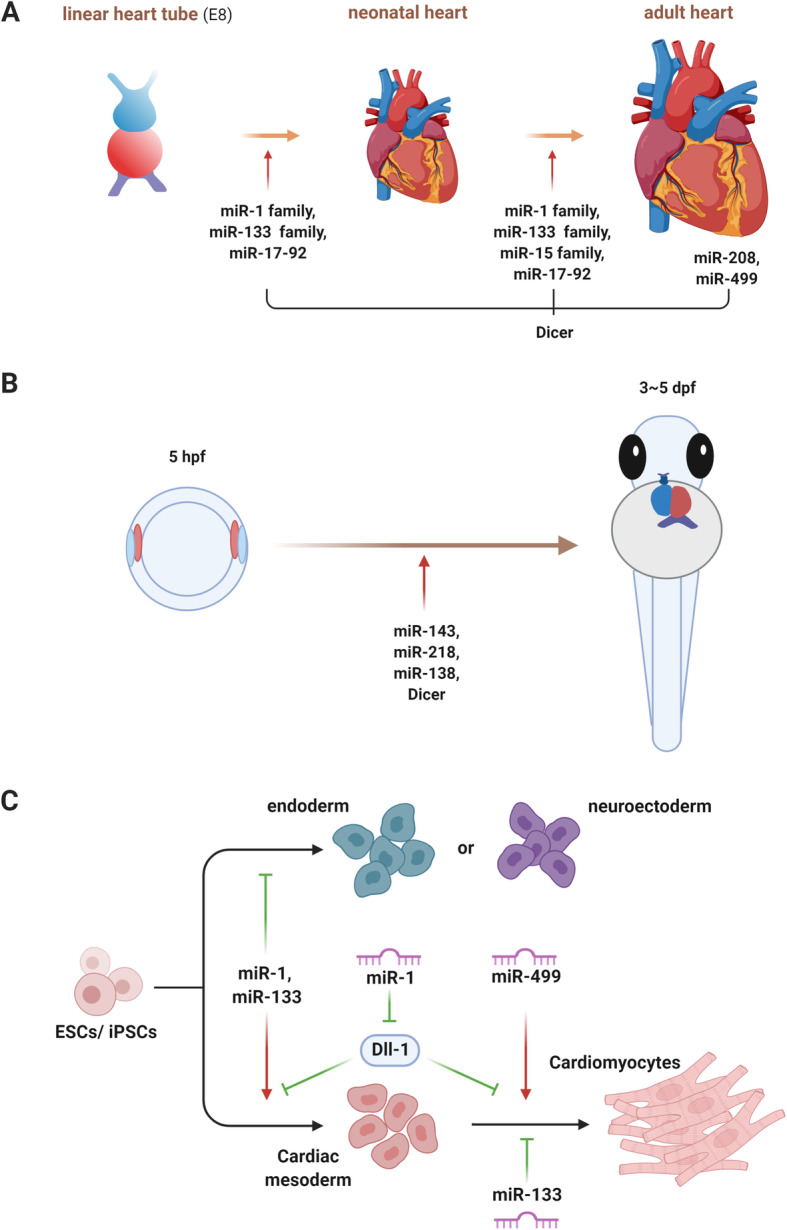
Summary of miRNAs regulating cardiac development. **a** miRNAs regulating embryonic cardiac development, postnatal growth, as well as adult heart homeostasis in mouse. **b** miRNAs regulating cardiac development in zebrafish. **c** miRNAs regulating cardiomyocyte differentiation from ESCs/iPSCs *in vitro*. hpf: hour post fertilization; dpf: day post fertilization; ESCs: embryonic stem cells; iPSCs: induced pluripotent stem cells.

In Fig. 1, we summarized the functions of Dicer and specific miRNAs important for cardiac development and growth in mouse, zebrafish and cardiomyocyte differentiation from ESCs (Fig. 1). Due to the close association between development and regeneration, these studies not only highlight the importance of the miRNA system in the heart, but also provide many valuable clues on how miRNAs regulate cardiomyocyte proliferation, and how miRNAs can be use as therapeutic tools to promote cardiac regeneration.

### microRNA and cardiomyocyte proliferation

In mammals, proliferation of cardiomyocyte is quickly diminished shortly after birth, and this process is a crucial aspect of the maturation of cardiomyocytes (Sadek and Olson 2020). However, the inability of mature cardiomyocytes to proliferation also render adult mammalian heart unable to regenerate after injury, which is one of the main reasons of negative prognosis of patients with myocardial infarction (Sadek and Olson 2020). Although multiple strategies have been proposed to regenerate the heart after injury (Sadek and Olson 2020), the approach of finding and utilizing potential cardiac stem cells or progenitor cells is no longer promising, as new research do not support the existence of such cardiac stem/progenitor cells with myogenic potentials (Li et al. 2018b; He et al. 2020). During both the regeneration of zebrafish heart (Poss et al. 2002) and neonatal mouse heart (Porrello et al. 2011b), proliferation of existing cardiomyocytes was found to be the major if not sole contributor of new cardiomyocytes. In addition, proliferation of adult cardiomyocytes, however minimal, is still present (Bergmann et al. 2009), and can be promoted (Sadek and Olson 2020). Therefore, promoting proliferation of endogenous cardiomyocytes is becoming increasingly attractive to not only scientists trying to understand cardiac biology, but also to physicians wishing to achieve cardiac regeneration in patients with heart attack (Sadek and Olson 2020).

As mentioned above, miRNAs are involved in modulating the heart development and various cardiac pathologies. During postnatal maturation of cardiomyocytes, the gene expression programs required for proliferation are gradually suppressed (Guo and Pu 2020). Hence it is not surprising that some miRNAs are involved in cardiomyocyte maturation by suppressing the expression of genes important for proliferation. And therapies targeting these microRNAs might be utilized to achieve cardiomyocyte proliferation and cardiac regeneration. On the other hand, miRNAs that can promote cardiomyocyte proliferation, which may or may not occur in a physiological scenario, can be directly used as therapies to promote cardiac regeneration.

Indeed, knockout of the muscle-specific miRNAs, including miR-1 (Zhao et al. 2005; Zhao et al. 2007; Wei et al. 2014) and miR-133 (Liu et al. 2008), led to increased cardiomyocyte proliferation *in vivo* during early postnatal stages (Zhao et al. 2007; Liu et al. 2008; Wei et al. 2014), and overexpression of miR-1 suppressed cardiomyocyte proliferation in embryonic heart (Zhao et al. 2005). Cell cycle related genes, such as *Ccnd1* (encoding cyclin D1) (Zhao et al. 2005; Zhao et al. 2007) and *Ccnd2* (encoding cyclin D2) (Liu et al. 2008) were found to be functional targets of miR-1 and miR-133 respectively. In addition, Porrello et al. found that expression of multiple members of the miR-15 family, which includes miR-15a, miR-15b, miR-16-1, miR-16-2, miR-195, and miR-497, are increased in mouse cardiac ventricles from postnatal day 1 to day 10 (Porrello et al. 2011a), among which miR-195 is the most up-regulated. miR-195 was found to induce mitotic arrest of cardiomyocytes (Porrello et al. 2011a), with its overexpression prematurely induced cardiomyocyte to exit cell cycle in neonatal mice. Several cell cycle genes, including *Chek1, Cdc2a, Birc5, Nusap1* and *Spag5,* were found to be targeted by miR-195 (Porrello et al. 2011a). In addition, inhibition of miR-15 family using locked-nucleic acid (LNA) modified anti-miR-15/16 can promote cardiomyocyte proliferation in postnatal mice (Porrello et al. 2011a). miR-15 family were also found to be upregulated in the infarct region after ischemic injury in both mice and pigs (Hullinger et al. 2012; Porrello et al. 2013), and injecting LNA anti-miR-15 in infarcted mouse heart protected against cardiac ischemic injury (Hullinger et al. 2012), although whether cardiomyocyte proliferation is affected was not examined in this study. Porrello et al. later performed similar experiments of inhibiting miR-15 before Ischemia Reperfusion (I/R) injury in adult mouse heart, and demonstrated both enhanced cardiomyocyte proliferation and recovered cardiac function (Porrello et al. 2013), confirming that miR-15 family inhibit cardiomyocyte proliferation. Porrello et al. also examined the effect of overexpressing miR-195 in infarcted neonatal mouse heart, and observed reduced cardiomyocyte proliferation and cardiac regeneration, which neonatal mouse heart is capable of after infarction (Porrello et al. 2013), further confirming that the suppressive role of miR-15 family in cardiomyocyte proliferation.

A few other microRNAs, whose expression are not specific or even enriched in the heart, have also been found to inhibit cardiomyocyte proliferation, after the initial characterization of the MyomiRs. In zebrafish, which can efficiently regenerate its injured heart, expression levels of miR-99/100 and Let-7a/c were found to be decreased during heart regeneration (Aguirre et al. 2014). Injecting miR-99/100 mimics blocked cardiac regeneration, while injecting antagomir against miR-99/100, which inhibits their function (Krutzfeldt et al. 2005), promoted cardiomyocyte proliferation and cardiac growth (Aguirre et al. 2014). Although adult murine heart cannot regenerate, miR-99/100 and Let-7a/c are well conserved in zebrafish and mammals, and suppression of miR-99/100 and Let-7a/c using adeno-associated virus 9 (AAV9) overexpressing anti-miRs can also bring the myocardial tissue to a partially dedifferentiated proliferative state after cardiac injury, and promote cardiac regeneration (Aguirre et al. 2014), highlighting the conservation of miRNAs and the molecular program they regulate. Besides *smarca5* and *fntb* identified as functional targets of miR-99/100 and Let-7a/c in zebrafish (Aguirre et al. 2014), *Ccnd2* and *E2f2* , two core components of cell cycle, were also found to be targets of let-7i-5p (Hu et al. 2019), emphasizing the direct blocking of cell cycle program by Let-7 family miRNAs in cardiomyocytes. Using zebrafish heart regeneration as a discovery platform, Beauchemin et al. also identified miRNA-101a as a negative regulator of cardiomyocyte proliferation and regeneration by targeting proto-oncogene *fosab* (*cfos*) in zebrafish (Beauchemin et al. 2015). However, despite targeting the same gene *c-Fos* in mouse, miRNA-101 was only found to inhibit murine cardiac fibroblast proliferation, thus regulating post-infarction fibrosis in mouse (Pan et al. 2012). Whether miRNA-101 regulates mammalian cardiomyocyte proliferation has not been demonstrated yet. miR-34, initially discovered as suppressor of cellular proliferation in cancer cells upon p53 activation (He et al. 2007), was not surprisingly, also found to inhibit cardiomyocyte proliferation (Yang et al. 2015). Delivery of miR-34a mimic blocks the cardiac regeneration of the neonatal mice (Yang et al. 2015), and injecting LNA anti-miR-34a improved post-MI remodeling in adult mice with increase cardiomyocyte proliferation (Yang et al. 2015). *Bcl2*, *CcnD1*, and *Sirt1* were found to be functional targets of miR-34a (Yang et al. 2015). Expression of miR-128 was found to be increased in the mouse heart during postnatal growth, and overexpressing miR-128 in neonatal mice impairs CM proliferation and cardiac function, while cardiac specific conditional knockout of miR-128 promotes CM proliferation (Yang et al. 2015; Huang et al. 2018). *Suz12*, which inhibits the inhibitor of cyclin-dependent kinase, p27, thus activating positive cell cycle regulators Cyclin E and Cdk2, was found to be targeted by miR-128, suggesting that miR-128 is also a crucial microRNA suppressing cardiomyocyte proliferation (Huang et al. 2018). miR-29a were found to be highly up-regulated in rat cardiomyocytes at the age of 4 weeks, and inhibition of miR-29a can relieve the suppression of *Ccnd2* and induce neonatal rat cardiomyocytes to reenter cell cycle through G1/S and G2/M transition (Cao et al. 2013). In addition, miR-216a was recently found to negatively regulate cardiomyocyte proliferation through inhibiting *Jak2* (Wang et al. 2019). However, the results of miR-29a and miR-216a were obtained in isolated rodent cardiomyocytes, and whether they regulate cardiomyocyte proliferation *in vivo* is still unknown. We summarized the miRNAs inhibiting cardiomyocyte proliferation in Table 1, with details of research models, major findings, identified targets, and relevant references.

**Table 1:** MicroRNAs inhibiting cardiomyocyte proliferation and cardiac regeneration

miRNA	Experimental models	Major findings	Targets	Ref.
miR-1	β-MHC-miR-1 transgenic mice	Overexpression of miR-1 decreases the number of cycling cardiomyocytes, results in developmental arrest at E13.5, secondary to thin-walled ventricles and heart failure.	*Hand2*, *Ccnd1*	(Zhao et al. 2005; Zhao et al. 2007; Wei et al. 2014; Gan et al. 2019)
	miR-1-2^−/−^ mice	miR-1-2^−/−^ mutants display thickening of the walls of the heart, while the increased weight may be due to hyperplasia.		
	miR-1 dKO mice	miR-1 dKO neonatal mice display proliferating cardiomyocytes.		
	neonatal mouse CMs	miR-1 represses cardiomyocyte G1/S phase transition.		
miR133a	miR-133a double-mutant mice	miR-133a double-mutant mice display late embryonic or neonatal lethality due to VSDs, while the surviving mutant mice display severe deficits in cardiac contractility and die from heart failure and sudden death. The absence of miR-133a expression results in ectopic expression of smooth muscle genes in the heart and aberrant cardiomyocyte proliferation.	*Srf* and *Ccnd2*	(Liu et al. 2008)
miR-15 family (miR-195, miR-15)	βMHC-miR-195 transgenic mice	Overexpression of miR-195 in the embryonic heart causes ventricular hypoplasia and ventricular septal defects.	*Chek1,* *Cdc2a,* *Birc5,* *Nusap1,* *Spag5*	(Porrello et al. 2011a; Hullinger et al. 2012; Porrello et al. 2013)
	delivery of anti- miR-15 family (LNA- miR-15 family) to neonatal mice	Post-natal inhibition of miR-15 family induces cardiomyocyte proliferation, as well as cardiomyocytes displaying disorganized sarcomeric structures.		
	neonatal CMs	Inhibition of miR-15 induces cardiomyocyte viability in response to hypoxia		
	delivery of miR-15 anti-miRs in hearts of both mice and pigs	Inhibition of miR-15 family protectes against cardiac ischemic injury		
	delivery of anti- miR-15 family (LNA- miR-15 family) to neonatal mice before MI	Inhibition of the miR-15 family induces cardiomyocyte proliferation and improves left ventricular systolic function.		
miR-29a	H9c2 cell line	Overexpression of miR-29a suppresses proliferation of H9c2 cell line.	*CCND2*	(Cao et al. 2013)
miR-99/100 and Let-7a/c	adult zebrafish	Injecting miR-99/100 mimics blocks cardiac regeneration, and injecting antagomir against miR-99/100 promots cardiomyocyte proliferation and cardiac growth	*smarca5* and *fntb*	(Aguirre et al. 2014)
	a murine model of MI	Inhibition of miR-99/100 and Let-7a/c induces the myocardial tissue to a partially dedifferentiated proliferative state after cardiac injury.		
miR-101a	adult zebrafish	Inhibition of miR-101a levels at the onset of cardiac injury enhances CM proliferation, while prolonged inhibition of miR-101a activity stimulates new muscle synthesis but with defects in scar tissue clearance.	*fosab* (*cfos*)	(Beauchemin et al. 2015)
miR-34a	delivery of miR-34a mimic to the myocardium at the time of MI	Overexpression of miR-34a inhibits functional post-MI recovery in neonatal mouse hearts.	*Bcl2*, *Ccnd1*, and *Sirt1*	(Yang et al. 2015)
	delivery of anti-miR-34a (LNA-34a) following MI in adult mice	Inhibition of miR-34a improves cardiac function in adult hearts post-MI.		
miR-128	cardiacspecific miR-128 overexpression mice	Overexpression of miR-128 impairs cardiac homeostasis, and inhibits neonatal cardiac regeneration.	*Suz12*	(Huang et al. 2018)
	cardiac-specific miR-128 knockout mice	Inhibition of miR-128 promotes adult cardiac regeneration.		
Let-7i-5p	neonatal mouse CMs	Let-7i-5p negatively regulates cardiomyocyte proliferation	*Ccnd2* and *E2f2*	(Hu et al. 2019)
	Ad-let-7i-5p/ AAV9-anti-let-7i-5p was delivered to mice	Overexpression of Let-7i-5p inhibits cardiomyocyte proliferation while inhibition of Let-7i-5p promotes cardiomyocyte proliferation.		
miR-216a	neonatal mouse CMs	miR-216a negatively regulates cardiomyocyte proliferation	*Jak2*	(Wang et al. 2019)

Exit of cell cycle is a hallmark of mammalian postnatal cardiomyocyte, thus the miRNAs involved in this process, negatively regulating cardiomyocyte proliferation, were identified and studied relatively early, almost as soon as mammalian miRNAs were annotated (Lagos-Quintana et al. 2002; Zhao et al. 2005). Although the inhibitory role of miRNAs in cardiomyocyte proliferation were abundantly demonstrated (Zhao et al. 2005; Zhao et al. 2007; Liu et al. 2008; Porrello et al. 2011a; Hullinger et al. 2012; Cao et al. 2013; Porrello et al. 2013; Aguirre et al. 2014; Beauchemin et al. 2015; Yang et al. 2015; Huang et al. 2018; Hu et al. 2019; Wang et al. 2019), miRNAs that may play a positive role in cardiomyocyte proliferation were not identified until 2012, when Eulalio et al. performed a high-throughput functional screening to identify miRNAs that trigger cardiomyocyte proliferation using a whole-genome miRNA library (Eulalio et al. 2012). 40 miRNAs were identified to increase karyokinesis and cytokinesis in both neonatal mouse and rat cardiomyocytes without inducing hypertrophy (Eulalio et al. 2012). miR-590-3p and miR-199a-3p were found to be the most effective microRNAs promoting cardiomyocyte proliferation (Eulalio et al. 2012), and both microRNAs can also significantly increase the proliferation of cardiomyocytes isolated from 7-days-old rats, which are postmitotic cardiomyocytes with extremely low rate of proliferation (Eulalio et al. 2012). What’s more, both microRNAs were found to increase cardiomyocyte proliferation *in vivo* and induce cardiac regeneration after myocardial infarction in mice, when overexpressed using AAV9 based delivery system (Eulalio et al. 2012). Another high-content screening of miRNAs on proliferation of human induced pluripotent stem cell-derived cardiomyocytes (hiPSC-CMs), using a updated whole-genome human miRNA library (Diez-Cuñado et al. 2018) identified 96 miRNAs that significantly increased both DNA synthesis and cytokinesis of hiPSC-CMs. Intriguingly, the effective miRNAs in this screening on human cardiomyocytes overlapped only minimally with the previously screening on rodent cardiomyocytes, and neither miR-590-3p and miR-199a-3p showed significant effect on hiPSC-CM proliferation (Eulalio et al. 2012; Diez-Cuñado et al. 2018), highlighting the evolutionary divergence of miRNA regulation between species, and calling for caution of translational application of miRNAs that showed promising results in rodents.

A few targets of miR-199a-3p and miR-590-3p were identified in the original screen (Eulalio et al. 2012). For example, *Homer1*, which was shown to regulate calcium signaling, and *Hopx*, which is involved in suppressing embryonic cardiomyocyte proliferation, were found to be targeted by miR-590-3p (Eulalio et al. 2012). And miR-199a-3p were found to directly target *Clic5*, a known inhibitor of cell proliferation (Eulalio et al. 2012). Other genes such as *Cd151* were also found to be functional targets of miR-199a-3p to promote cardiac regeneration (Tao et al. 2019). However, subsequent studies from both groups which performed the rodent and human cardiomyocyte screens showed that the miRNA hits from both screens appeared to mainly target a common Hippo-YAP pathway to regulate cardiomyocyte proliferation (Diez-Cuñado et al. 2018; Torrini et al. 2019).

The Hippo signaling pathway, which consists of several conserved upstream kinases and downstream effector transcription factors, controls organ growth and size through regulating cell proliferation (Yu and Guan 2013). In mammals, the receptors at the upstream of the Hippo signaling pathway sense the growth inhibition signal and undergo a series of phosphorylation reactions of kinases that eventually phosphorylate the downstream effector transcription factors Yap and Taz, thus retain Yap and Taz in the cytoplasm (Zheng and Pan 2019). Cytoskeleton is an important upstream regulator of Hippo-Yap signaling, as depolymerization of F-actin inhibits the phosphorylation of Hippo kinases and traps Yap/Taz from entering the nucleus, while polymerized F-actin triggers nuclear localization of Yap/Taz (Zheng and Pan 2019). Once in the nucleus, Yap/Taz activate transcriptions of their targets genes, which normally induce cell proliferation (Yu and Guan 2013). Loss of upstream kinases of the Hippo pathway induces hypoplastic growth of the developing heart (Heallen et al. 2011), while loss of Yap, the major Hippo effector in cardiomyocytes, impairs cardiac regeneration in neonatal mice (Xin et al. 2013), highlighting the importance of this pathway on cardiomyocyte proliferation.

Several miRNAs hits from the rodent screen (Eulalio et al. 2012), including miR-199a-3p, miR-302d, miR-373, and miR-590-3p, all target *Stk38l* and *Lats1-2*, which encode upstream kinases of the Hippo pathway (Torrini et al. 2019). In addition, some microRNA hits (Eulalio et al. 2012) also suppress expression of cardiomyocyte cytoskeleton genes, leading to actin polymerization, which in turn releases Yap to enter the nucleus to activate proliferation. *Cofilin2* (*Cfl2*), a regulator of actin polymerization enriched in cardiomyocyte, was found to be a major functional cytoskeletal gene targeted by microRNA hits including miR-199a-3p, and its downregulation alone could trigger robust cardiomyocyte proliferation (Torrini et al. 2019). Besides miR-199a-3p, quite a few other hits in the rodent screen (Eulalio et al. 2012), namely miR-1825, miR-302d, miR-373 and miR-33b*, were all found to be directly or indirectly targeting *Cofilin2* (Torrini et al. 2019). In addition, miR-199a-3p, the most effective microRNA promoting rodent cardiomyocyte proliferation, directly targets multiple upstream Yap inhibitory kinases, including *Taok1-3*, *Stk38l*, and *Btrc*, which encoding β-TrCP that catalyzes ubiquitination of phosphorylated Yap (Torrini et al. 2019). The multiple levels of inhibition of Yap by miR-199a-3p may explain its strongest pro-proliferation effect. Intriguingly, in the human microRNA screen, despite minimal overlap of hits with the rodent screen, 67 of the 96 miRNAs that induce cardiomyocytes proliferation were found to target components of the Hippo-YAP pathway (Diez-Cuñado et al. 2018), and mRNAs encoding most Hippo/YAP pathway components in hiPSC-CMs were found to be present in RISC complex, suggesting they were indeed physically targeted by miRNAs (Diez-Cuñado et al. 2018).

In addition to the miRNAs identified from the two screens, an independent research also found that multiple microRNAs target Hippo-YAP signaling pathway to promote cardiomyocyte proliferation. Tian et al. showed that miR-302–367 cluster can target multiple upstream kinases of the Hippo pathway to activate YAP, thus inducing cardiomyocyte proliferation (Tian et al. 2015), and the transient expression of miR-302–367 mimics could improve cardiac function after injury, while long-term expression of miR-302–367 induced cardiac dysfunction (Tian et al. 2015). In conclusion, the discovery of different miRNAs in different mammalian species targeting a common signaling pathway (Hippo-Yap) to regulate cardiomyocyte proliferation indicates that the signaling pathways are more conserved than miRNAs targeting them, and highlights the core position of Hippo-YAP signaling in regulating cardiomyocyte proliferation.

Besides genes encoding components of the Hippo pathway, other genes, especially cell cycle related genes, have recently been found to be targeted by various miRNAs promoting cardiomyocyte proliferation. miR-17-92 cluster, known for its oncogenic function (He et al. 2005), and importance for cardiac development (Wang et al. 2010; Chen et al. 2013), was found to induce CM proliferation in embryonic, postnatal and adult hearts (Chen et al. 2013). Knockout of the entire cluster led to decreased perinatal cardiomyocyte proliferation, fewer cardiomyocyte in the postnatal heart, and smaller heart overall (Chen et al. 2013). Conditional overexpressing of the cluster in early and late embryonic heart, using *Nkx2.5-Cre* and *Myh6-Cre* respectively, led to increased cardiomyocyte proliferation (Chen et al. 2013), and induced overexpression of the cluster in adult cardiomyocyte using *Myh6-CreERT2* also enhanced proliferation of the cardiomyocyte and protected the heart from MI injury with smaller scars (Chen et al. 2013). miR-19, a member of miR-17-92 cluster, was found to be the most potent miRNA in the cluster in inducing cardiomyocyte proliferation (Chen et al. 2013), and PTEN, a well-known tumor suppressor, was found to be directly targeted by miR-19 (Chen et al. 2013). Interestingly, an independent study found that miR-17-3p, another member of the miR-17-92 cluster, can promote cardiomyocyte proliferation as well as hypertrophy by directly targets PTEN and indirectly targets TIMP-3 (Shi et al. 2017), both in cultured murine cardiomyocytes and in a mouse model of excise-induced cardiac hypertrophy. miR-210 was found to be highly expressing in mouse HL-1 cardiomyocytes and downregulated after hypoxic insult (Hu et al. 2010), and injecting non-viral minicircles overexpressing miR-210 into infarcted murine heart showed protection against cardiomyocyte apoptosis and cardiac function (Hu et al. 2010), thus it was first identified as a pro-survival miRNA. miR-210’s role in cardiomyocyte proliferation was revealed in a subsequent study, in which miR-210 showed pro-proliferation activity on adult rat cardiomyocytes (Arif et al. 2017), and its transgenic overexpression led to a significant increase in CM proliferation after myocardial infarction (Arif et al. 2017) *Adenomatous polyposis coli* (*APC*), a core component of Wnt signaling pathway that is a suppressor of proliferation, was identified as the direct functional target of miR-210 (Arif et al. 2017). miR-294 was found to be highly expressed in embryonic mouse heart and its expression is quickly diminished after birth (Borden et al. 2019), and it showed pro-proliferation activity both *in vitro*, in isolated neonatal rat ventricular myocytes, and *in vivo*, as AAV9-mediated overexpression of miR-294 in the infarcted mouse heart promoted cardiomyocyte cell cycle reentry and functional recovery of the heart (Borden et al. 2019). Wee1, an suppressor of Cyclin B1/CDK1 complex and mitosis, was found to be directly targeted by miR-294 (Borden et al. 2019). miR-1825 was found to induce proliferation of isolated adult rat cardiomyocytes and was shown to target multiple genes regulating mitochondria mass, including *Ndufa10* encoding a mitochondrial electron transporter (Pandey et al. 2017). Intriguingly, miR-1825 was also found to increase the expression of miR-199a, the strongest miRNA promoting rodent cardiac regeneration (Pandey et al. 2017). Therefore, it is not surprising AAV9-mediated overexpression of miR-1825 showed strong induction of cardiomyocyte proliferation and cardiac regeneration after MI (Pandey et al. 2017).

There are reports of other miRNAs stimulating cardiomyocytes proliferation, while lacking evidence from myocardial infarction model which is still currently the golden standard of assessing cardiac regeneration in mammals. miR-708, which is enriched in neonatal cardiomyocyte, was shown to promote proliferation of neonatal rat ventricular myocytes (Deng et al. 2017). Delivered using neutral lipid emulsion (NLE) to adult mouse heart stressed with isoproterenol, miR-708 was shown to protect the heart from hypertrophy and fibrosis and promote function recovery of the heart (Deng et al. 2017). However, no evidence of cardiomyocyte proliferation *in vivo* was demonstrated in this study, despite that *Mapk14*, an MAP kinase associated with cell cycle, was identified as a target of miR-708 (Deng et al. 2017). miRNA-204, shown to increase proliferation of both neonatal and adult rat cardiomyocytes *in vitro* (Liang et al. 2015), was also found to promote embryonic and adult mouse cardiomyocyte proliferation *in vivo* when overexpressed driven by a cardiomyocyte specific promoter (Liang et al. 2015). *Jarid2*, an epigenetic factor that is required for cardiac development (Lee et al. 2000), and was known for inhibiting embryonic cardiomyocyte proliferation (Toyoda et al. 2003), was identified as a functional target of miRNA-204 (Liang et al. 2015). Without a MI model to test miRNA-204’s ability to cardiac regeneration (Liang et al. 2015), this study warranted more research on the therapeutical capacity of miRNA-204. miR-31a-5p was identified in a miRNA array to be upregulated in postnatal day 10 cardiomyocytes and was found to promote neonatal rat cardiomyocyte proliferation by targeting *RhoBTB1*, encoding a Rho small GTPase (Xiao et al. 2017). Knocking down miR-31a-5p using antagomirs injected intraperitoneally led to decreased cardiomyocyte proliferation *in vivo*, but experiments of overexpressing miR-31a-5p after MI were not performed (Xiao et al. 2017), thus making it difficult to evaluate the therapeutical potential of miR-31a-5p. In addition, miR-25, which was found to be reduced post MI and I/R injury (Qin et al. 2019), showed pro-proliferation activity in neonatal rat cardiomyocytes and adult human cardiomyocytes in culture, as downregulation of miR-25 by anti-miR could reduce proliferation, and *Bim* was found to be one of the functional targets of miR-25 (Qin et al. 2019). A subsequent study found that miR-25 can promote proliferation of hiPSC-CM *in vitro* and zebrafish cardiomyocytes in *vivo* (Wang et al. 2020), and identified *FBXW7*, a cell-cycle regulatory factor that mediates the ubiquitin-dependent proteolysis of many positive cell-cycle regulators (Welcker and Clurman 2008), as a direct target gene of miR-25 (Wang et al. 2020). However, miR-25 was found to be targeting *Serca2* to impair cardiac contractility *in vivo* (Wahlquist et al. 2014), and whether it regulates cardiomyocyte proliferation *in vivo* is yet to be experimentally investigated. A recent study found that miR-152 is transcriptionally controlled by Yap, and can promote isolated mouse neonatal cardiomyocyte proliferation by targeting cell cycle genes *Cdkn1b* and *Dnmt1* (Wang et al. 2018). In contrast, a study on miR-152’s function *in vivo* revealed that it regulates mitochondria iron homeostasis and is associated with heart failure (LaRocca et al. 2020), without evidence of proliferation affected. We summarized the miRNAs promoting cardiomyocyte proliferation in Table 2, with details of research models, major findings, identified targets, and relevant references.

**Table 2:** MicroRNAs promoting cardiomyocyte proliferation and cardiac regeneration

miRNA	Experimental models	Major findings	Targets	Ref.
miR-590-3p	neonatal rat CMs, neonatal mouse CMs, postnatal rat (P7) CMs	Overexpression of miR-590-3p increases CM proliferation.	*Homer1*, *Hopx*, Hippo pathway	(Eulalio et al. 2012; Torrini et al. 2019)
	delivery of hsa-miR-590-3p complexed with a lipid transfection reagent into the heart of neonatal rats	The left ventricle walls of the hearts appeared markedly thicker with increases CM proliferation.		
	delivery of AAV9-miR590 precursor miRNAs into the neonatal mice	At 12 days after injection, the hearts were morphologically normal, but significantly enlarged.		
	delivery of AAV9-miR590 into the adult mice after infarction	The infarct size was significantly reduced in mice.		
miR-199a-3p	neonatal rat CMs, neonatal mouse CMs, postnatal rat (P7) CMs	Overexpression of miR-199a-3p increases CM proliferation.	*Homer1*, *Clic5*, *Cd151*, Hippo pathway	(Eulalio et al. 2012; Lesizza et al. 2017; Gabisonia et al. 2019; Tao et al. 2019; Torrini et al. 2019)
	delivery of hsa-miR-199a-3p complexed with a lipid transfection reagent into the heart of neonatal rats	The left ventricle walls of the hearts appeared markedly thicker with increases CM proliferation.		
	delivery of AAV9-miR-199a-3p precursor miRNAs into the neonatal mice	At 12 days after injection, the hearts of these animals were morphologically normal, but significantly enlarged.		
	delivery of AAV9-miR-199a-3p into the adult mice after infarction	The infarct size was significantly reduced in mice.		
	delivery of AAV9-miR 199a-1 pri-miRNA into the infarcted pig hearts	The treated animals showed marked improvements in both global and regional contractility, increased muscle mass and reduced scar size. At longer follow-up, however, persistent and uncontrolled expression of the microRNA resulted in sudden arrhythmic death of most of the treated pigs.		
miR-17-92 Cluster	miR-17-92-cKO mice	miR-17-92 is sufficient to induce cardiomyocyte proliferation in embryonic and postnatal hearts.	*Pten*	(Chen et al. 2013)
	cardiac-specific overexpression miR-17-92 transgenic mice	Overexpression of miR-17-92 induces cardiomyocyte proliferation in embryonic, neonatal and adult hearts. Overexpression of miR-17-92 in adult cardiomyocytes protects the heart from myocardial infarction-induced injury.		
	neonatal rat CMs	miR-17-92 is sufficient to induce neonatal cardiomyocyte proliferation.		
	direct injection of miR-19a/19b mimics into the heart of a mouse model of MI	miR-19a/19b overexpression enhances cardiomyocyte proliferation and stimulates cardiac regeneration in response to myocardial infarction (MI) injury.		
miRNA-204	neonatal and adult rat CMs	Overexpression of miRNA-204 promotes cardiomyocyte proliferation	*Jarid2*	(Liang et al. 2015)
	cardiac-specific overexpression miRNA-204 transgenic mice	Overexpression of miRNA-204 promotes cardiomyocyte proliferation throughout the embryonic and adult stages.		
miR302–367 cluster	cardiac-specific overexpression miR302–367 transgenic mice	Overexpression of miR302–367 promotes cardiomyocyte proliferation in embryonic and postnatal hearts, and miR302–367 promotes adult cardiac regeneration after myocardial infarction.	Hippo pathway	(Tian et al. 2015)
	delivery miR302–367 mimics into the adult mice	Overexpression of miR302–367 promotes cardiac regeneration and improves function after injury.		
miR-210	neonatal rat CMs	miR-210 induces proliferation.	*Apc*	(Arif et al. 2017)
	miR-210 overexpressing transgenic (210-TG) mice	miR-210 overexpression promotes CM proliferation in adult mice post-ischemic injury.		
miR-708	H9C2 cells, neonatal rat CMs, neonatal mouse CMs	Overexpression of miR-708 promotes myocardium regeneration and heart function recovery.	*Mapk14*	(Deng et al. 2017)
	delivery miR-708 mimics to a mice model of cardiac hypertrophy			
miR-1825	adult CMs	Overexpression of miR-1825 induces robust proliferation.	Hippo pathway	(Pandey et al. 2017)
	direct injection of AAV-miR-1825 into mice	Overexpression of miR-1825 improves heart function.		
miR-31a-5p	neonatal rat CMs	Overexpression of miR-31a-5p promotes cardiomyocyte proliferation.	*Rhobtb1*	(Xiao et al. 2017)
	neonatal rats were injected intraperitoneally with the miR-31a-5p antagomir	Inhibition of miR-31a-5p decreases cardiomyocyte proliferation.		
miR-294	neonatal and adult rat CMs	Overexpression of miR-294 promotes cell cycle activity.	*Wee1*	(Borden et al. 2019)
	delivery of AAV-9-miR-294 to mice after MI	Overexpression of miR-294 promotes cell cycle reentry and improves cardiac function.		
miR-152	neonatal CMs	Overexpression of miR-152 promotes neonatal cardiomyocyte proliferation.	*Cdkn1b* and *Dnmt1*	(Wang et al. 2018)
miR-25	neonatal and adult CMs	Inhibition of miR-25 reduces neonatal and adult cardiomyocyte proliferation.	*Bim* and *FBXW7*	(Qin et al. 2019; Wang et al. 2020)
	hESC-CM	miR-25 promotes hESC-CM proliferation.		
	Transgenic zebrafish	Overexpressing miR-25 promotes cardiomyocyte proliferation in zebrafish.		

Interestingly, recent research discovered that long non-coding RNAs (LncRNAs), some of which function as sponge of miRNAs, also play a role in cardiomyocyte proliferation and regeneration (Braga et al. 2020). For example, cardiomyocyte regeneration-related lncRNA (CRRL) was found to inhibit cardiomyocyte proliferation *in vitro* and *in vivo* (Chen et al. 2018), and loss of CRRL improved cardiac repairs in neonatal and adult rats post-MI (Chen et al. 2018). More importantly, miR-199a-3p, the potent activator of cardiomyocyte proliferation, was found to be suppressed by CRRL, which serves as a sponge for miR-199a-3p (Chen et al. 2018). There are other LncRNAs that were also associated with cardiomyocyte proliferation, such as CPR (Cardiomyocyte Proliferation Regulator) (Ponnusamy et al. 2019), CAREL (cardiac regeneration-related lncRNA) (Cai et al. 2018), and AZIN2-sv (AZIN2 splice variant) (Li et al. 2018a). The details of their molecular function and relevance to cardiac regeneration can be find in other great reviews (Braga et al. 2020), as miRNAs are the focus of this review.

**Fig. 2:**
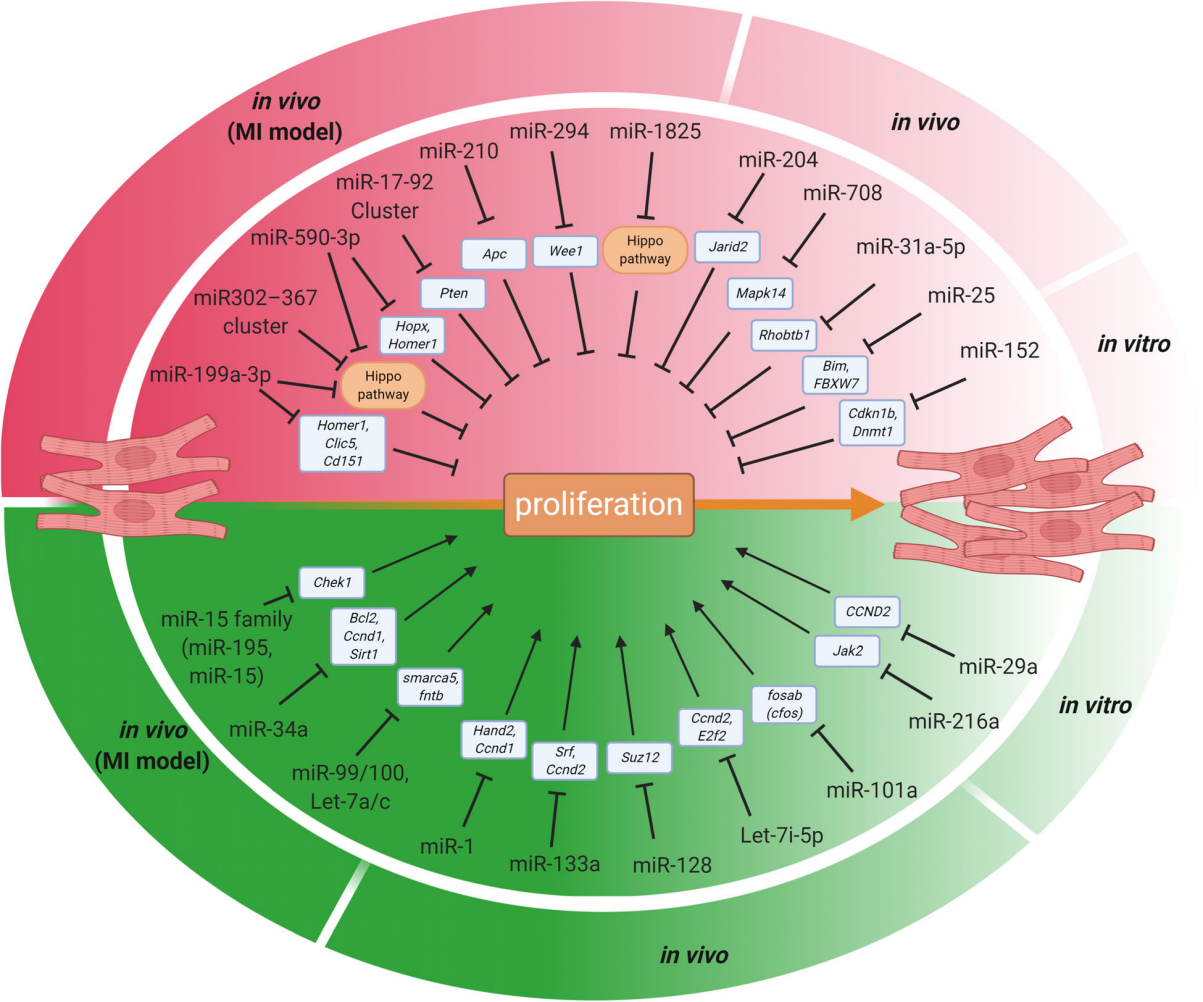
Summary of miRNAs regulating heart regeneration. Different miRNAs and their targets stimulating (red area) or inhibiting (green area) cardiomyocyte proliferation and heart regeneration were shown. miRNAs are grouped according to the amount of evidence supporting their function: (1) miRNAs with evidence regulating heart regeneration *in vivo* (MI model) and cardiomyocyte proliferation *in vitro*; (2) miRNAs with evidence regulating cardiomyocyte proliferation both *in vivo* (without MI model) and *in vitro*; (3) miRNAs with only *in vitro* results of regulating cardiomyocyte proliferation. These three groups were indicated by different depth of red and green colors. MI: myocardial infarction

We now know many miRNAs can positively or negatively regulate cardiomyocyte proliferation (Table 1, Table 2, and Fig. 2). As more miRNAs are identified and more investigation of their function in cardiomyocytes proceed, the number of microRNAs regulating cardiomyocyte proliferation will likely increase in the coming years. Our knowledge on miRNA regulating cardiomyocyte proliferation and cardiac regeneration will be constantly updated, which might unveil other core regulators of cardiomyocyte proliferation, like the Hippo pathway, and will provide an expanding arsenal of microRNAs for further pre-clinical and clinical exploration aiming at human cardiac regeneration. However, caution should be taken in evaluating the pharmaceutical values of a certain miRNA, as some miRNAs only showed pro-proliferation activity *in vitro* (Wang et al. 2018; Qin et al. 2019), and some has *in vivo* evidence without a cardiac disease model like MI or I/R injury, which is more relevant to cardiac regeneration (Xiao et al. 2017). More importantly, most evidence of miRNAs regulating cardiomyocyte proliferation was acquired from rodent models, except for the miRNA screen on hiPSC-CMs (Diez-Cuñado et al. 2018), which showed alarmingly little overlap of positive hits with the miRNA screen on rodent cardiomyocytes (Eulalio et al. 2012). The discrepancy strongly suggests that careful and extensive pre-clinical trials are needed, and efficacy and molecular mechanism must be confirmed in human cardiomyocytes, before these promising miRNAs are applied to human patients.

### miRNA and cardiomyocyte reprogramming

The efficacy of generating new CMs from preexist-CMs through proliferation is still low, despite promising methods including the ones using microRNA can enhance this process. Cardiac fibroblasts, which are abundant in the heart after cardiac insults, emerged in the last few years as a possible source of new CMs. Fibroblasts not only can be reprogrammed into induced pluripotent stem (iPS) cells (Takahashi and Yamanaka 2006), but also can be reprogrammed into other functional cell types, including cardiomyocytes (Qian and Srivastava 2013). Several reprogramming strategies have been reported in recent years (Qian and Srivastava 2013), most of which were based on cardiac specific transcription factors GATA4, MEF2C and TBX5 (Qian et al. 2012). Interestingly, miRNAs were also shown to be capable of directly inducing cellular reprogramming of fibroblasts to cardiomyocyte both *in vitro* and *in vivo* (Jayawardena et al. 2012). Jayawardena et al. identified the combination of muscle-specific miRNA: miR-1, miR-133, miR-208, and miR-499, could mediate the reprogramming of cardiac fibroblasts to cardiomyocyte-like cells *in vitro* (Jayawardena et al. 2012). After transfected cardiac fibroblasts with these miRNAs, the expression of myocyte-specific markers, as well as spontaneous calcium transients and electrophysiology demonstrated that the reprogrammed cells have the characteristic of a cardiomyocyte-like phenotype (Jayawardena et al. 2012). *in vivo* study also showed direct conversion of cardiac fibroblasts to cardiomyocytes *in situ* after injecting the four microRNAs following myocardial injury (Jayawardena et al. 2012). A follow-up study used the combination of the same miRNAs to reprogram primary human atrial cardiac fibroblasts into cardiomyocytes, and (Paoletti et al. 2020), and about 10% of the cells were reprogrammed into cardiomyocyte-like cells with expression of cardiac Troponin T and spontaneous calcium transient (Paoletti et al. 2020). These results further proved the powerful effect of miRNAs in cardiac regeneration, by repurposing the cells in the injured heart to be regenerative. However, the overall cardiac reprogramming strategy faces several challenges in fundamental biology as well as clinical translation. First, the efficiency of reprogramming is generally low, and dependent on the age of the fibroblasts to be reprogrammed (Mahmoudi et al. 2019). The resulting induced cardiomyocytes often lack a mature phenotype of adult cardiomyocytes (Paoletti et al. 2020). Therefore, their cellular and electrical coupling with surrounding cells, as well as their survivability and possible arrhythmogenic effects need to be carefully examined, and their functional maturation may be required before therapeutic applications (Sadek and Olson 2020). Finally, the delivery of a cocktail of factors, even if they are small miRNAs, is difficult. Currently, open-chest direct injection can ensure relative local expression of the cocktail miRNAs, but less invasive and more efficient methods for delivery might need to be optimized in large animal models before clinical applications. More detailed information on cardiac reprogramming can be find in reviews covering this topic (Sadahiro and Ieda 2020; Sadek and Olson 2020).

### Therapeutic application of miRNA in cardiac regeneration

Due to their small size and relative pleiotropic effects, microRNAs have been considered promising candidates to be therapeutically delivered to treat human cardiac diseases. And several approaches have been made to explore the safety and efficacy of microRNA treatment *in vivo*.

Direct transgenic overexpression of microRNAs is often robust and consistent (Chen et al. 2013), while it is not desirable for therapeutic usage as it permanently alter the genome of the recipients. AAV-based delivery system, which does not integrate into the genome, has been proven efficient to induce microRNA expression and cardiac regeneration in multiple rodent experiments (Eulalio et al. 2012; Borden et al. 2019; Gao et al. 2019). For example, Eulalio et al. confirmed the AAV9-based delivery system stably expressed hsa-miR-590 and hsa-miR199a in mouse heart, which significantly increased CM proliferation *in vivo* without any sign of CM hypertrophy (Eulalio et al. 2012). However, the requirement to express the pre-microRNA in the AAV9 system prohibits specific expression of a single mature microRNA, thus compounding the overall therapeutic effect, and the long term persistent of the AAV9 vector in the transduced CM may also lead to undesired consequences (Tian et al. 2015; Braga et al. 2020). For instance, miR302–367 was shown to promote adult cardiac regeneration, however, the mice exhibited compromised heart function after prolonged miR302–367 overexpression (Tian et al. 2015).

Directly injecting microRNA mimics or anti-miRNAs directly to the myocardium, or targeting them to the myocardium after intravenous injection, may avoid these potential issues. However, it requires protection against nuclease degradation *in vivo*, ability to cross endothelial barrier, efficient entrance to cardiomyocytes, and minimal toxicity. And significant progress has been made to optimize the formulating of miRNA delivery system (Braga et al. 2020). Lipid-based delivery system, originated from lipofection technology of entrapping small nucleic acid with cationic lipid or neutral lipid molecules (Kulkarni et al. 2018), has been validated for efficient delivery of miRNAs *in vivo* by multiple experiments in rodent (Tian et al. 2015; Deng et al. 2017; Lesizza et al. 2017; Gao et al. 2019). The systems based on cationic lipids, such as Lipofectamine RNAiMAX, are optimized for transfection efficiency, while the systems based on neutral lipids, such as MaxSuppressor RNALancerII, are better at maintaining stability in circulation (Braga et al. 2020). Both systems based on cationic lipids (Lesizza et al. 2017; Gao et al. 2019) and neutral lipids (Tian et al. 2015; Deng et al. 2017; Gao et al. 2019) showed efficient delivery. In particular, Gao et al. compared the two methods in parallel experiments, and slightly higher miRNA expression was detected in Lipofectamine RNAiMAX based system, while both methods yield similar functional outcome (Gao et al. 2019). It is also worth noting that a single dose of injection of the most potent microRNA of activating CM proliferation, miR-199a-3p, was sufficient to induce robust cardiac regeneration in mice, highlighting the effectiveness of both the microRNA and the lipid-based delivery method (Lesizza et al. 2017). One significant drawback of these lipid base delivery systems is that the particle size is relatively large (>1μm) and may lead to toxicity and inflammation *in vivo* (Kulkarni et al. 2018). Newer systems utilizing ionizable cationic lipids can entrap miRNAs in the cationic form, make smaller particles (<100 nm), and become neutral under physiological pH, thus generating neutral lipid nanoparticles (LNPs) (Kulkarni et al. 2018), which has been used in 2018 to deliver siRNA against Transthyretin in clinical trials (Adams et al. 2018). LNPs have not been tested for miRNA delivery for cardiac regeneration yet. Their safety and efficiency tested in other disease models, especially human clinical trials, may provide evidence for its usage in cardiac regeneration.

What’s more, biocompatible injectable hydrogels have also been tested for their efficiency in helping microRNA delivery (Pandey et al. 2017; Wang et al. 2017; Yang et al. 2019). Both hyaluronic acid-based (Wang et al. 2017; Yang et al. 2019) and gelatin/silicate-based hydrogels (Pandey et al. 2017) showed promising capacities in facilitating miRNA uptake in cardiomyocytes *in vivo*. In addition, biological carriers such as bacterial minicells (MacDiarmid and Brahmbhatt 2011) and exosomes (Jiang et al. 2017) are both exciting new tools for miRNA delivery *in vivo*, and we may witness their application in cardiac regeneration in the near future.

Thanks to the recent advance in material science and nano-technology, delivering methods of miRNAs are constantly updated. Given that most *in vivo* trials were done in small animals, which is relatively easy for miRNA delivery, and whose cardiac physiology as well as cardiomyocyte biology are quite different from human, testing in large animals will be required before moving on to therapeutic applications.

A recent study indeed tested microRNA therapy in swine myocardial infarction model, which is more relevant to human heart disease. AAV6 was used to express the most potent microRNA promoting rodent cardiomyocyte proliferation, miR-199a, in the infarcted pig hearts (Gabisonia et al. 2019). One month after myocardial infarction, a marked reduction in scar size can be observed and the global cardiac function was recovered in the AAV6-miR-199a treated animals (Gabisonia et al. 2019). Unfortunately, long-term expression of AAV6-miR-199a led to sudden death of most of the pigs, possibly due to arrhythmias as recorded by the subcutaneously implanted miniaturized recorder (Gabisonia et al. 2019). This symptom might be the consequence of the expression of miR-199a-5p, which is generated from miR-199a-1 pri-miRNA and is known to have adverse effects on the heart (Song et al. 2010; el Azzouzi et al. 2013; Zhang et al. 2016; Li et al. 2017). Another possible reason could be that large numbers of cardiomyocyte undergoing dedifferentiation disrupted their electrophysiology, causing lethal arrhythmias (Gabisonia et al. 2019). As the first test of miRNA in cardiac regeneration in large animals, this pioneering work shed light on the promising future of miRNA therapy, while also emphasized the unknowns and risks of this approach (Gabisonia et al. 2019).

## Conclusions and future outlook

As one of the most important organs in our body, the heart has been and will be a major focus of basic and translational research. Studying how it forms and works will help us understand its diseases and find ways to treat them. In the last few decades, microRNA is emerging to be an important regulator of virtually all biological processes in mammals, and its involvement in cardiac development, diseases and regeneration has been gradually discovered, and its potential to be utilized in therapeutics has gained attraction due to its small size and pleiotropic effects. One key characteristic of cardiomyocytes in mammals is that most of them cease to proliferate after birth, and will not regeneration after cardiac injury in adults, thus leaving cardiac regeneration the greatest challenge in treating cardiac diseases. microRNAs have been demonstrated to be important in this process, as some cardiac specific miRNAs required for mature phenotypes of the cardiomyocytes inhibit proliferation, while other miRNAs promote cardiomyocyte proliferation (Braga et al. 2020).

As most microRNAs suppress gene expression to achieve their cellular function, the research on miRNA regulating cardiac regeneration could surely advance our understanding of the core regulators of CM proliferation (Fig. 2). It is not surprising that multiple positive regulators of cell cycle are targets of miRNAs enriched in cardiomyocyte that suppress proliferation (Zhao et al. 2007; Cao et al. 2013; Huang et al. 2018), and negative regulators of cell cycle are targets of miRNAs promoting cardiomyocyte proliferation (Chen et al. 2013; Wang et al. 2018; Borden et al. 2019) (Fig. 2). However, it is intriguing that many miRNAs promoting CM proliferation found by different research groups converge on the Hippo-YAP signaling pathway (Tian et al. 2015; Diez-Cuñado et al. 2018; Torrini et al. 2019) (Fig. 2), despite little overlap of miRNAs that target this pathway between human and rodents (Diez-Cuñado et al. 2018). This indicates that Hippo-YAP signaling pathway is fundamental for cardiomyocyte proliferation, and signaling pathway and transcription factors are more conserved than miRNAs targeting them. In fact, the convergence demonstrated that miRNAs can be used as probes to dissect conserved core mechanisms of a biological process and may be applied to explore mechanisms of other systems.

Another important outcome of the research of miRNAs regulating CM proliferation is the therapeutic application of miRNAs in treating cardiac diseases. The relatively small size of miRNAs makes them easy to deliver, and the short seed sequence of miRNAs gives them the ability to target and regulate multiple genes, thus the most pleiotropic miRNAs can be selected for further therapeutic development. However, the multiplex nature of the targeting mechanism of miRNA also guarantees inevitable side effect, and the real overall safety and efficacy of a therapeutic miRNA have to be thoroughly examined in animal models before moving on to human. Although a variety of studies have demonstrated promising regenerative efficacy of several miRNAs in cardiac injury models of small animals, only miR-199a-3p has been reported to improve cardiac function in preclinical studies of large animals, while its long-term expression led to lethal cardiac arrest (Gabisonia et al. 2019). On one hand, this highlights the importance of preclinical trials on large animals to ensure the safety and efficacy of miRNA therapy, as they are more relevant to human than mice. On the other hand, it emphasizes the necessity to further our understanding of the mechanism of cardiomyocyte proliferation as well as miRNAs regulating this process, to design better strategies. A clinically feasible treatment for cardiac diseases must produce significant improvement while reduce the adverse effects to the minimum at the same time, especially when it is targeting a vital organ like the heart. Better delivery system, in which the dose, duration, target cell specificity, and toxicity can be tightly controlled, might be able to minimize the possibly unavoidable side effects of miRNA, thus enhancing the safety of the therapy. With constantly updating delivery system for small nucleic acid, especially new lipid nanoparticles, as well as biological carriers such as bacterial minicells and exosomes, better strategy of miRNA therapy may be available in the near future. Moreover, different miRNAs may constitute a complex network to modulate the cardiac development and regeneration by targeting different genes and pathways, thus indicating that a cocktail strategy might be able to optimize the outcomes of the miRNA treatment.

With decades-long basic research on miRNA and cardiac development and regeneration, scientists now are moving on to carry out exciting translational research on miRNA therapies promoting cardiac regeneration. The accumulating knowledge will help us design better therapeutic strategies which may eventually lead to novel clinical applications to treat ischemic heart diseases. Major obstacles in the translational research have their roots in the unknowns of the biology, as well as the lack of suitable technologies. Therefore, comprehensive research on the detailed mechanisms of how miRNA regulating cardiomyocyte proliferation, as well as development of new delivery technologies, will be required before successful translational studies in preclinical and clinical settings.

## Data Availability

.

## Abbreviations

miRNA(s): microRNA(s)
MI: myocardial infarction
CM(s): cardiomyocyte(s)
ESC(s): embryonic stem cell(s)
iPSC(s): inducible pluripotent stem cell(s)
pri-miRNA: primary miRNA
pre-RISC: pre-RNA-induced silencing complex
MRF: myogenic regulatory factor
dKO: double knockout
α-MHC: α-myosin heavy chain
β-MHC: β-myosin heavy chain
TR: thyroid hormone receptor
THRAP1: TR-associated protein 1
Dll-1: Delta-like 1
*dmiR-1*: *Drosophila miR-1*
hESC-CM(s): human embryonic stem cells derived adult ventricular cardiomyocyte(s)
I/R: ischemia reperfusion
*Ccnd1*: cyclin D1
*Ccnd2*: cyclin D2
*Chek1*: checkpoint kinase 1
AAV: adeno-associated virus
LNA: locked-nucleic acid
hiPSC-CM(s): human induced pluripotent stem cell-derived cardiomyocyte(s)
*Cfl2*: cofilin2
*Adenomatous polyposis coli*: *APC*
CRRL: Cardiomyocyte regeneration-related lncRNA
CPR: Cardiomyocyte Proliferation Regulator
CAREL: cardiac regeneration-related lncRNA
AZIN2-sv: AZIN2 splice variant
LNP: Lipid Nanoparticle
